# Think Outside the Lyme Box: Optic Neuropathy and Polyarthritis Caused by Borrelia miyamotoi

**DOI:** 10.7759/cureus.95879

**Published:** 2025-11-01

**Authors:** Ken Nagahata, Takeshi Ikeda, Yoshihisa Tsuji

**Affiliations:** 1 Department of Rheumatology and Clinical Immunology, Sapporo Medical University, Sapporo, JPN; 2 Department of General Medicine, Sapporo Medical University, Sapporo, JPN; 3 Department of Ophthalmology, Sapporo Medical University, Sapporo, JPN

**Keywords:** borrelia miyamotoi, lyme disease, optic neuropathy, polyarthritis, tick-borne disease

## Abstract

*Borrelia miyamotoi* disease (BMD) is an emerging tick-borne infection that can cause a range of symptoms. While typically presenting as an acute febrile illness, less common but significant complications, such as optic neuropathy, can occur. We describe a 68-year-old man who presented with high fever, rash, acute vision loss in the left eye, and polyarthralgia following a tick bite. Investigations confirmed BMD with associated optic neuropathy and polyarthritis. This case highlights the importance of considering BMD in patients with compatible symptoms and tick exposure and underscores the potential for severe ophthalmological and joint involvement.

## Introduction

*Borrelia miyamotoi* disease (BMD) is an emerging tick-borne infectious disease caused by the spirochete *Borrelia miyamotoi*, transmitted by the bites of *Ixodes* ticks [1]. This pathogen belongs to the relapsing fever group of Borrelia but is transmitted by the same *Ixodes* ticks that transmit Lyme disease [2]. Since the first human case was reported in Russia in 2011, BMD has been recognized as an important public health issue, particularly in the Northern Hemisphere [2]. The clinical course of BMD is often similar to that of Lyme disease, making it a crucial differential diagnosis [3].

The typical clinical presentation of BMD is an acute febrile illness characterized by symptoms such as headache, myalgia, arthralgia, and occasionally erythema migrans [1]. Laboratory findings often include thrombocytopenia, leukopenia, and elevated liver enzymes, mimicking anaplasmosis [4]. While many cases resolve with appropriate treatment, more severe manifestations can occur. Neurological complications, including meningoencephalitis, have been reported [3,5], often in immunocompromised patients [6,7]. Among these, optic neuropathy is a rare but serious complication that can lead to chronic and irreversible vision loss, as has been reported in Europe [3].

Here, we present the case of a 68-year-old man who developed two significant complications concurrently: optic neuropathy leading to acute vision loss and imaging-confirmed polyarthritis. This case highlights the potential for BMD to cause severe, multi-organ inflammatory manifestations and underscores the importance of considering this diagnosis in patients with compatible symptoms and a history of tick exposure.

## Case presentation

A 68-year-old man presented with a two-week history of high fever (over 39°C), generalized erythema, and polyarthralgia. One week prior to the presentation, he developed acute pain and vision loss in his left eye. During this period, he received a seven-day course of levofloxacin at a previous clinic without improvement. He recalled being bitten by a tick on the left forearm approximately six weeks before his symptoms began. His past medical history was notable for synovitis, acne, pustulosis, hyperostosis, and osteitis (SAPHO) syndrome, for which he had been receiving methotrexate for the past 10 years.

**Figure 1:**
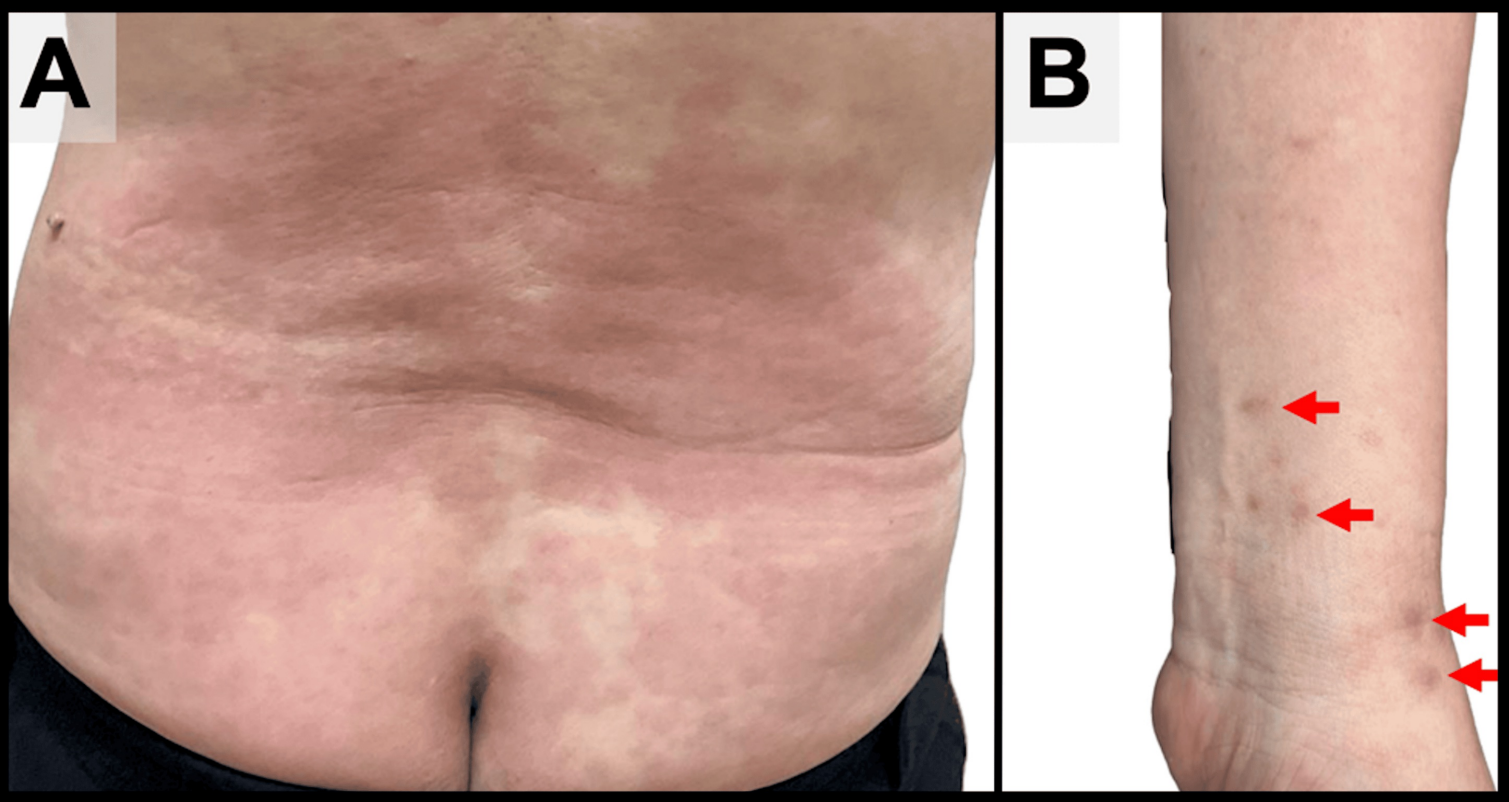
(A) Erythema on the trunk. (B) Visible tick bite marks on his left forearm.

**Figure 2:**
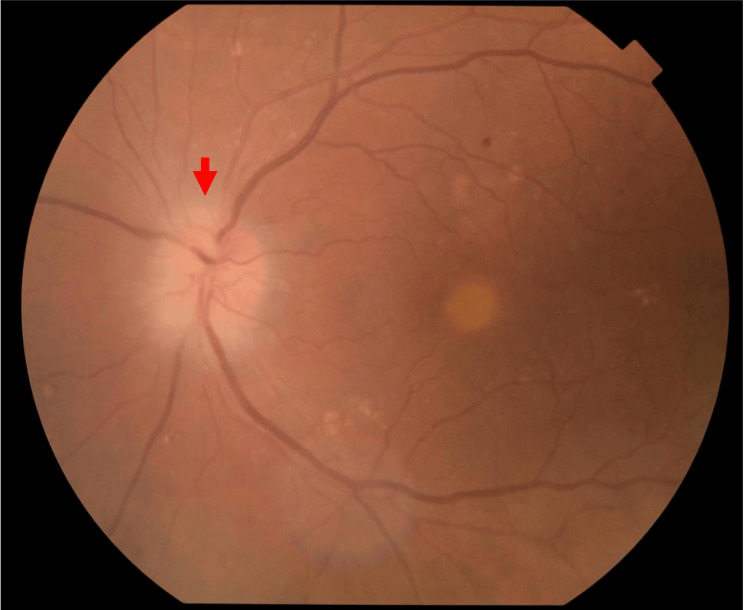
The fundus examination revealed left optic disc edema (red arrow).

**Figure 3:**
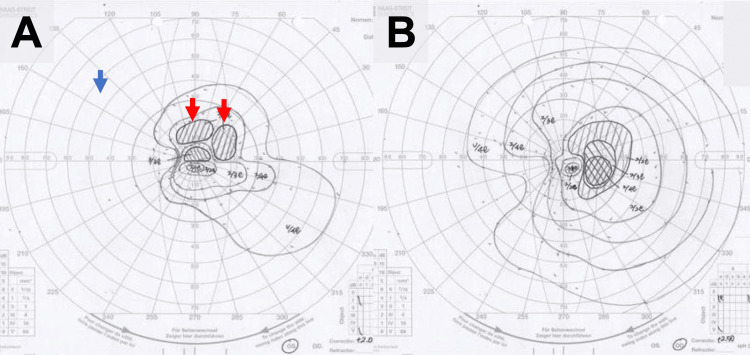
Findings in the (A) left eye and the (B) right eye. The ophthalmoscopic image of the left eye shows optic disc edema (red arrows), and a corresponding visual field chart demonstrates a temporal defect (blue arrow). The right eye is unremarkable.

**Figure 4:**
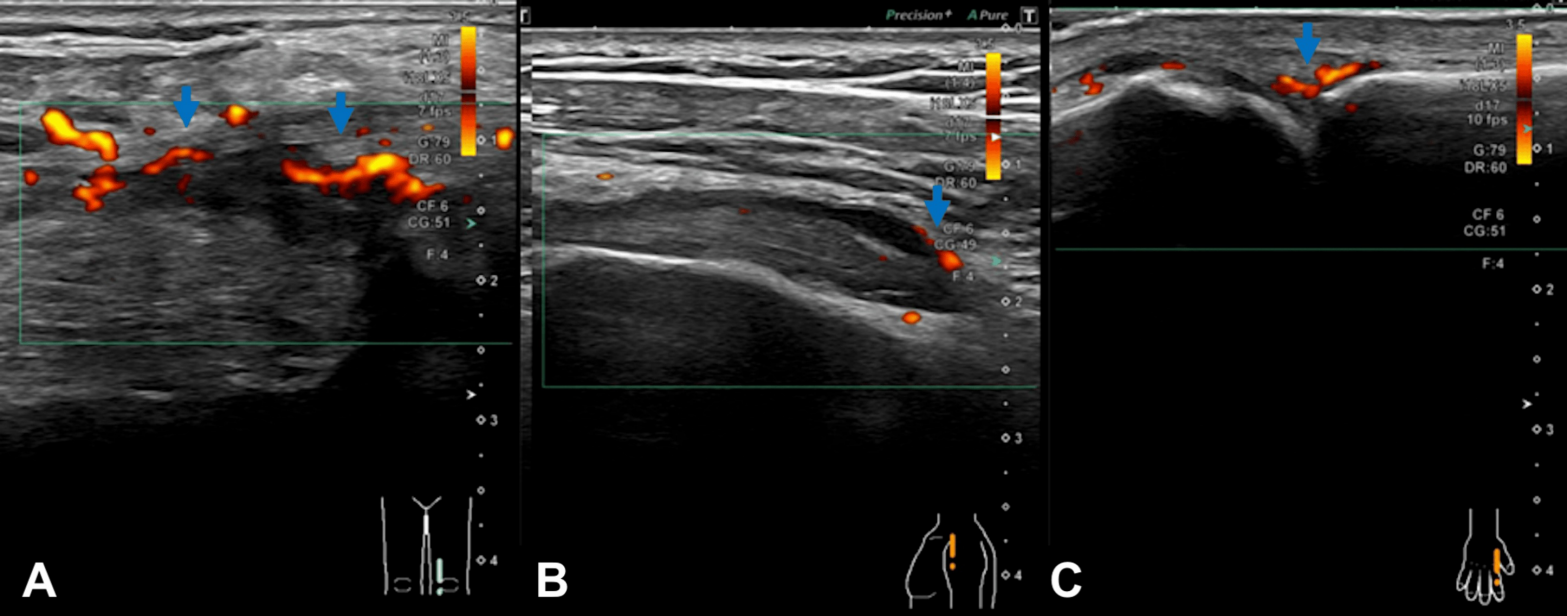
Ultrasonography of the (A) left knee, (B) shoulder, and (C) metacarpophalangeal joints demonstrating synovitis with high-power Doppler signals (blue arrows), a representative finding of the patient's polyarthritis.

On physical examination, he had diffuse erythema on his extremities and trunk, with visible tick bite marks on his left forearm (Figure 1). An ophthalmological examination of the left eye revealed a relative afferent pupillary defect (RAPD) and decreased visual acuity to 20/100. Funduscopic examination showed optic disc edema (Figure 2), and perimetry confirmed a temporal visual field defect (Figure 3). Musculoskeletal ultrasonography demonstrated polyarthritis affecting both shoulders, knees, and metacarpophalangeal joints (Figure 4).

**Figure 5:**
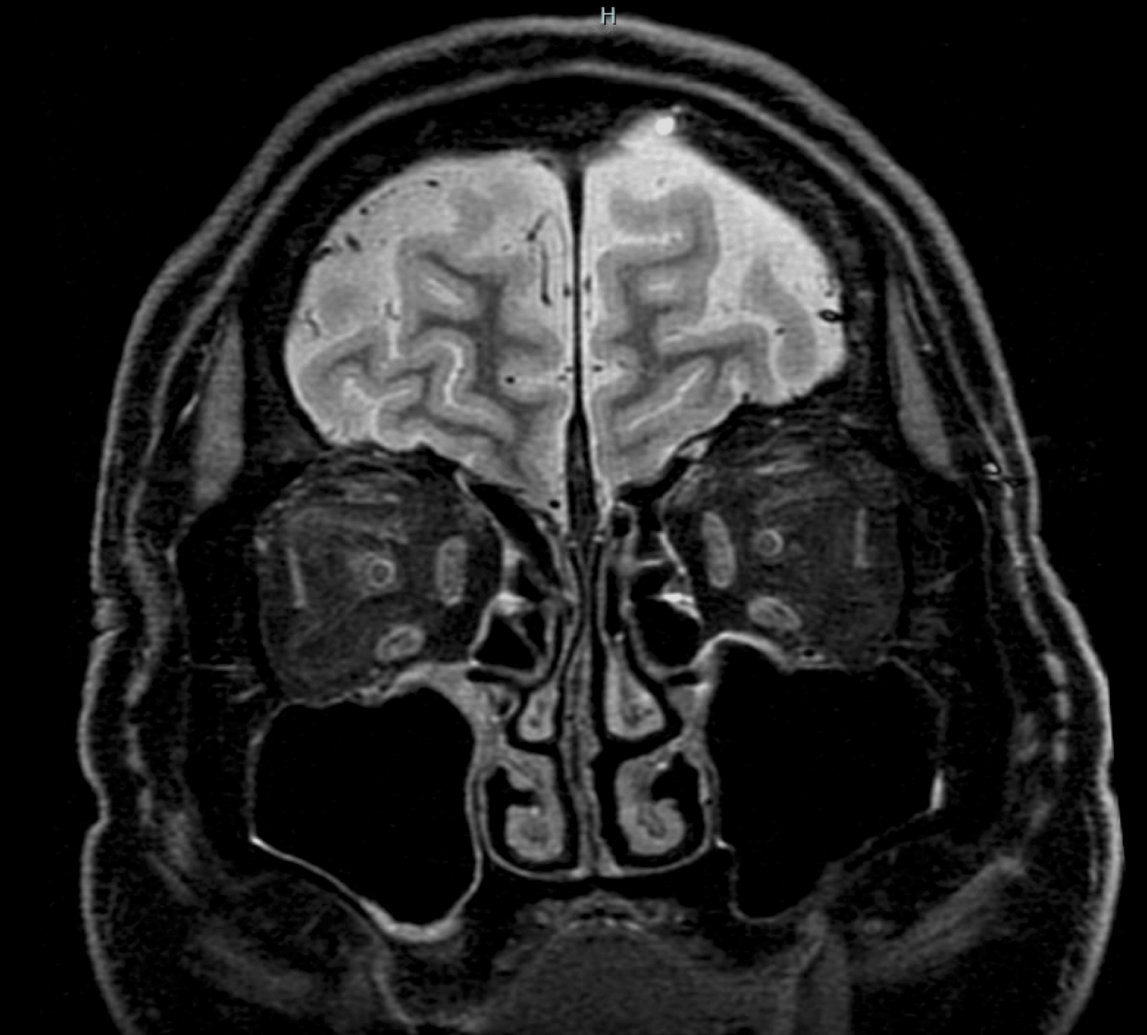
Brain MRI, non-contrast T1-weighted imaging, showed no significant abnormalities, such as optic nerve signal hyperintensity.

Laboratory investigations showed an elevated white blood cell count of 13,900/μL, a highly elevated C-reactive protein level of 19 mg/dL, thrombocytopenia (89,000/μL), and mildly elevated liver enzymes (Table 1). Serum samples were positive for both IgM and IgG antibodies for BMD by Western blot. However, a whole blood polymerase chain reaction (PCR) for the pathogen was negative. Serology and PCR for *Borrelia burgdorferi* (Lyme disease) were negative, as were antibodies against Yezo virus. Brain magnetic resonance imaging (MRI) showed no significant abnormalities, including the absence of optic nerve signal intensity, suggesting optic neuropathy rather than optic neuritis (Figure 5). A cerebrospinal fluid (CSF) examination was not performed due to the patient's refusal.

**Table 1:** Laboratory results ALP: Alkaline phosphatase, ALT: Alanine aminotransferase, APTT: Activated partial thromboplastin time, AST: Aspartate aminotransferase, BUN: Blood urea nitrogen, Ca: Calcium, Cl: Chloride, CRP: C-reactive protein, IgG: Immunoglobulin G, IgM: Immunoglobulin M, K: Potassium, LD: Lactate dehydrogenase, MCV: Mean corpuscular volume, Na: Sodium, PT-INR: Prothrombin time, γGT: Gamma-glutamyl transpeptidase

Parameter	Patient Value	Normal Range
White blood cell	13.9	3.3–8.6×10^3^/μL
Segmented neutrophil	60	45–68%
Lymphocyte	18	26–43%
Eosinophil	9	0–10%
Hemoglobin	12.5	13.7–16.8 g/dL
MCV	94.5	83.6–98.2 fL
Platelet count	8.9	15.8–34.8 ×10^4^/μL
Total protein	7.4	6.6–8.1 g/dL
Albumin	2.9	4.1–5.1 g/dL
Total bilirubin	0.7	0.4–1.5 mg/dL
Creatine kinase	23	59–248 U/L
AST	26	13–30 U/L
ALT	56	10–42 U/L
LD	247	124–222 U/L
ALP	70	38–113 U/L
γGT	70	13–64 U/L
Creatinine	0.95	0.65–1.07 mg/dL
Uric acid	4.2	3.7–7.0 mg/dL
BUN	12	8–20 mg/dL
Na	139	138–145 mmol/L
Cl	106	101–108 mmol/L
K	4.1	3.6–4.8 mmol/L
Ca	8.7	8.8–10.1 mg/dL
CRP	19	0.00–0.14 mg/dL
PT-INR	1.14	0.85–1.15
APTT	29.3	26.9–38.1 sec
Fibrinogen	511	200–400 mg/dL
D-dimer	1.3	<1.0 μg/mL
IgM antibody for Borrelia miyamotoi	positive	negative
IgG antibody for Borrelia miyamotoi	positive	negative
IgM antibody for Borrelia burgdorferi	negative	negative
IgG antibody for Borrelia burgdorferi	negative	negative
IgM antibody for Yezo virus	negative	negative
IgG antibody for Yezo virus	negative	negative

Based on the clinical presentation, tick exposure history, and positive serology, a diagnosis of BMD with associated optic neuropathy and polyarthritis was made. Initially, intravenous ceftriaxone was recommended, but the patient refused this treatment. Therefore, he was subsequently treated with oral doxycycline 200 mg/day for 21 days, based on the first-line empirical outpatient treatment for Lyme disease. His fever resolved, and hematological abnormalities improved; however, the vision loss in his left eye and his polyarthritis persisted without improvement at the end of the antibiotic course. The patient was subsequently transferred to a local clinic for follow-up, so we have been unable to track their progress.

## Discussion

This case is of clinical importance, as it describes a severe presentation of BMD characterized by the concurrent onset of optic neuropathy and polyarthritis, two uncommon complications, particularly in an immunosuppressed patient.

While the differential diagnosis of acute visual impairment and polyarthritis includes systemic diseases such as sarcoidosis and Behçet's disease [8], eliciting a history of a tick bite was the crucial clue that led to suspicion of a tick-borne illness. This is particularly relevant for clinicians in the Northern Hemisphere, where *B. miyamotoi* is widely distributed in ticks. The pathogen prevalence in ticks (tick prevalence) was highest in Asia, at 2.8% for *Ixodes persulcatus*. This rate is higher than the prevalence observed in North America (*Ixodes scapularis*: 1.1% and *Ixodes pacificus*: 0.7%) and Europe (*Ixodes ricinus*: 1.0%), suggesting a potentially higher risk of BMD in Asia [5].

The diagnosis in this case was established serologically, as the blood PCR test was negative. This discrepancy is common in the diagnostic course of BMD. PCR detects the spirochete's DNA directly, which is typically present in the blood only briefly during the early, acute phase of infection (spirochetemia) [4]. Given that our patient presented two weeks into his illness and had already received antibiotics, it is not surprising that the pathogen's DNA was no longer detectable in his blood. In contrast, serology detects the host's antibody response, which develops later in the course of the illness and persists, explaining why his antibody tests were positive.

However, it is crucial to recognize the limitations of serology in the very early stages of infection. As it takes time for the body to produce a detectable level of antibodies, initial tests can be negative. Indeed, a large case series reported that as few as 16% of patients with acute BMD were seropositive at their initial presentation [4], highlighting the limited utility of serology in the earliest phase of the disease. Therefore, clinicians should have a high degree of suspicion for BMD based on clinical and epidemiological clues, even if initial serology is negative. In such cases, diagnosis often relies on demonstrating a significant rise in antibody titers in paired serum samples collected several weeks apart.

Severe neurological complications like meningoencephalitis are known consequences of BMD, especially in immunocompromised hosts [6]. The pathophysiology of optic neuropathy, a rare complication of BMD, is thought to be complex.

Direct central nervous system (CNS) invasion by the spirochete is a plausible mechanism, supported by a previous report detecting *B. miyamotoi* DNA in the CSF [3]. At the same time, immune-mediated mechanisms are likely critical. These include "molecular mimicry," where *Borrelia* antigens resemble host neural components [9], and secondary neurological damage resulting from an overproduction of inflammatory cytokines [10]. In this case, the absence of optic nerve signal hyperintensity on MRI helped differentiate this condition from typical demyelinating optic neuritis, suggesting an alternative pathophysiology such as ischemia or direct non-inflammatory microbial damage.

While arthralgia is a common symptom of BMD, polyarthritis with ultrasonographic evidence of synovitis is less frequently documented [5]. The presentation of early-onset polyarthritis in our case differs from the typical late-stage mono- or oligoarthritis of Lyme arthritis, suggesting that BMD may exhibit a different pattern of articular manifestations. The mechanism may involve the deposition of immune complexes (antigen-antibody complexes) in the synovium, a process also proposed for Lyme arthritis [11].

In this case, the patient's clinical improvement was limited despite a 21-day course of doxycycline. This unfavorable outcome may be attributable to the delay in initiating appropriate therapy, but also potentially to a more severe disease course secondary to an immunosuppressed state induced by prior immunosuppressive therapy. Currently, there are no established treatment guidelines specifically for BMD; therapy is empirically based on that for Lyme disease, which typically involves a 14-day course of doxycycline for acute illness [1,2,6]. However, for CNS complications such as meningitis, intravenous antibiotics with good CSF penetration, such as ceftriaxone, or a longer duration of therapy may have been necessary. Although the patient's refusal of inpatient treatment limited the therapeutic options, this experience underscores the importance of timely diagnosis and the selection of appropriate antimicrobial therapy tailored to disease severity. Furthermore, it is possible that by the time of diagnosis, irreversible structural damage to the optic nerve had already occurred, limiting the potential for recovery even with appropriate antimicrobial therapy.

This report has several limitations. First, a CSF examination was not performed; therefore, we could not definitively prove direct CNS invasion. Second, as this is a single case report, its findings cannot be generalized. Nevertheless, this case illustrates the diverse clinical spectrum of BMD and emphasizes the significant challenges in its diagnosis and management.

## Conclusions

BMD is an emerging infection that can cause severe complications like optic neuropathy and polyarthritis, particularly in immunosuppressed individuals. Clinicians in tick-endemic regions should consider BMD for patients with fever, rash, and unexplained neurological or rheumatological symptoms, especially after a tick bite. Prompt diagnosis and treatment are crucial to prevent irreversible sequelae.
